# Real-time changes in brain activity during tibial nerve stimulation for overactive bladder: Evidence from functional near-infrared spectroscopy hype scanning

**DOI:** 10.3389/fnins.2023.1115433

**Published:** 2023-04-05

**Authors:** Xunhua Li, Rui Fang, Limin Liao, Xing Li

**Affiliations:** ^1^Department of Urology, China Rehabilitation Research Center, School of Rehabilitation, Capital Medical University, Beijing, China; ^2^University of Health and Rehabilitation Sciences, Qingdao, China; ^3^Department of Occupational Therapy, China Rehabilitation Research Center, Beijing, China; ^4^China Rehabilitation Science Institute, Beijing, China

**Keywords:** overactive bladder, tibial nerve stimulation, fNIRS, brain activity, central mechanism

## Abstract

**Purpose:**

To use functional near-infrared spectroscopy (fNIRS) to identify changes in brain activity during tibial nerve stimulation (TNS) in patients with overactive bladder (OAB) responsive to therapy.

**Methods:**

Eighteen patients with refractory idiopathic OAB patients were recruited consecutively for this pilot study. At baseline, all patients completed 3 days voiding diary, Quality-of-Life score, Perception-of-Bladder-Condition, and Overactive-Bladder-Symptom score. Then 4 region-of-interest (ROI) fNIRS scans with 3 blocks were conducted for each patient. The block design was used: 60 s each for the task and rest periods and 3 to 5 repetitions of each period. A total of 360 s of data were collected. During the task period, patients used transcutaneous tibial nerve stimulation (TTNS) of 20-Hz frequency and a 0.2-millisecond pulse width and 30-milliamp stimulatory current to complete the experiment. The initial scan was obtained with a sham stimulation with an empty bladder, and a second was obtained with a verum stimulation with an empty bladder. Patients were given water till strong desire to void, and the third fNIRS scan with a verum stimulation was performed. The patients then needed to urinate since they could not tolerate the SDV condition for a long time. After a period of rest, the patients then were given water until they exhibited SDV state. The fourth scan with sham fNIRS scan in the SDV state was performed. NIRS_KIT software was used to analyze prefrontal activity, corrected by false discovery rate (FDR, *p* < 0.05). Statistical analyses were performed using GraphPad Prism software; *p* < 0.05 was considered significant.

**Results:**

TTNS treatment was successful in 16 OAB patients and unsuccessful in 2. The 3 days voiding diary, Quality-of-Life score, Perception-of-Bladder-Condition, and Overactive-Bladder-Symptom score were significantly improved after TNS in the successfully treated group but not in the unsuccessfully treated group. The dorsolateral prefrontal cortex (DLPFC) (BA 9, Chapters 25 and 26) and the frontopolar area (FA) (BA 10, Chapters 35, 45, and 46) were significantly activated during TNS treatment with an empty bladder rather than with an SDV. Compared with the successfully treated group, the unsuccessfully treated group did not achieve statistical significance with an empty bladder and an SDV state.

**Conclusion:**

fNIRS confirms that TNS influences brain activity in patients with OAB who respond to therapy. That may be the central mechanism of action of TNS.

## Introduction

Overactive bladder (OAB) is characterized by urinary urgency, frequency, nocturia, and urgent incontinence in the absence of an infection or other evident disease by the International Continence Society (ICS) (Haylen et al., 2010). It affects numerous people, causing significant economic and quality of life problems (Stewart et al., 2003; Coyne et al., 2011; Reynolds et al., 2016). Treatment of OAB can be challenging, as many patients have persistent symptoms in spite of behavioral and oral pharmacologic therapies (Chancellor et al., 2014). Tibial nerve stimulation (TNS) is an alternative for those with OAB, and it comes in three forms: percutaneous (PTNS), implanted (ITNS), and transcutaneous (TTNS) (Schneider et al., 2015; Te Dorsthorst et al., 2020). Nonetheless, the precise mechanism of action in OAB therapy has yet to be determined.

Functional neuroimaging is useful in studying the brain micturition pathway (Fowler and Griffiths, 2010). According to functional neuroimaging studies, females with OAB had elevated afferent signaling to the cingulate, insular, and frontal cortices (Griffiths et al., 2005; Komesu et al., 2011). Several regions of the brain are essential for regular urination, and bladder filling also activates different brain regions (de Groat, 1998; Nardos et al., 2014; Griffiths, 2015). Studies using functional magnetic resonance imaging (fMRI) revealed higher activity in areas related with urine symptoms and urgency (Griffiths et al., 2007; Tadic et al., 2012). Functional near-infrared spectroscopy (fNIRS) has the benefits of noninvasive, portable, optic-based, and places little physical mobility limits to investigate the central micturition circuit (Duan et al., 2012; Geng et al., 2017). Furthermore, fNIRS has greater temporal resolution, can generate stable signals quicker, and can directly identify changes in oxyhemoglobin (HbO) signals in addition to deoxyhemoglobin (HbR) signals, making it superior to fMRI (Geng et al., 2017). Numerous fNIRS and fMRI studies have shown the accuracy and reproducibility of fNIRS signals, offering an evidential support for their use (Cui et al., 2011; Duan et al., 2012; Geng et al., 2017). In this study, we used fNIRS to study real-time brain activity during TNS treatment among OAB patients and explain the central mechanism of TNS.

## Materials and methods

### Patients

With Institutional Review Board approval (IRB:2021 N012), we recruited 18 women (mean age, 42.39 ± 19.72 years) with refractory idiopathic OAB who chose TTNS. The inclusion criteria were as follows: age 18 to 75 years, 72 h of recording urination with at least 8 voids every day and 7 days of abstaining from anticholinergic and β3 adrenergic receptor agonist prior to TTNS. Drug usage was unchanged throughout therapy. Patients with untreated symptoms of urinary tract infection, bladder tumor, or urinary stones were ineligible, as were those who were pregnant, had a pacemaker or implanted defibrillator, had combined renal insufficiency, Parkinson’s disease, complete spinal cord injury, mental illness that prevented them from cooperating with doctors, skin lesions at the treatment place, and had participated in other drug or device clinical trials within 1 month prior to enrollment.

### Stimulation procedures

Evaluations were not carried out when the subjects were having their periods. At the beginning of the study, every patient recorded their voiding diary for 72 h, received a score on their Quality of Life (QoL), assessed their Perception of Bladder Condition (PPBC), and completed an Overactive Bladder Symptom score (OABSS). If patients met the inclusion exclusion criteria, we then conducted the fNIRS trial on them. Patients were instructed on how to use the stimulator after the experiment, and they then went home to stimulate themselves. The stimulate parameters was as follows: 20-Hz frequency and a 0.2-millisecond pulse width and 30-milliamp stimulatory current. Patients performed TNS 1 h per day for 30 days and then returned to our facility to follow up and complete a 72-h voiding diary prior to the follow-up day as well as a Qol score, PPBC score, and OABSS. Clinical treatment success was characterized as either a decrease of daily frequency voids of at least 30% or a reduction of urgency voids of at least 50% (Cava and Orlin, 2022). Region-of-interest (ROI) fNIRS scans were showed in Table 1.

**Table 1:** Channel locations for the fNIRS cap.

Ch	MNI coordinates (*x y z*)			BA	Brain area	Probability
1	34.84	−9.26	69.78	6	Pre-motor and supplementary motor cortex	0.9
2	−42.88	−10.92	63.07	3	Somatosensory cortex	1
3	47.77	−13.74	61.09	3	Somatosensory cortex	1
4	38.29	15.42	58.52	6	Pre-motor and supplementary motor cortex	0.9
5	−43.63	13.35	53.61	6	Pre-motor and supplementary motor cortex	0.9
6	−53.42	−16.59	56.15	2	Somatosensory cortex	1
7	56.89	−23.36	52.98	40	Supramarginal gyrus	0.71
8	50.52	5.28	50.87	6	pre-motor and supplementary motor cortex	0.9
9	37.51	29.64	49.48	8	Includes frontal eye field	0.61
10	−42.72	27.26	44.72	8	Includes frontal eye field	0.61
11	−55.13	2.24	44.44	6	Pre-motor and supplementary motor cortex	0.9
12	−61.04	−26.06	46.01	40	Supramarginal gyrus	0.71
13	64.65	−25.85	41.39	40	Supramarginal gyrus	0.71
14	57.96	4.06	40.64	6	Pre-motor and supplementary motor cortex	0.9
15	46.1	30.82	39.25	8	Includes frontal eye field	0.61
16	28.13	50.72	36.07	8	Includes frontal eye field	0.61
17	−36.8	46.73	31.46	9	Dorsolateral prefrontal cortex	0.79
18	−51.78	24.48	33.06	9	Dorsolateral prefrontal cortex	0.79
19	−61.63	−2.81	34.68	6	Pre-motor and supplementary motor cortex	0.9
20	−65.04	−29.62	38.38	40	Supramarginal gyrus	0.71
21	63.37	−1.67	30.79	6	Pre-motor and supplementary motor cortex	0.9
22	53.06	28.62	29.94	9	Dorsolateral prefrontal cortex	0.79
23	40.07	49.89	25.65	9	Dorsolateral prefrontal cortex	0.79
24	20.2	63.73	23.77	9	Dorsolateral prefrontal cortex	0.79
25	−7.92	66.05	23.29	9	Dorsolateral prefrontal cortex	0.79
26	−26.4	60.8	21.4	9	Dorsolateral prefrontal cortex	0.79
27	−44.66	45.28	21.7	46	Dorsolateral prefrontal cortex	0.61
28	−55.98	21.56	24.68	9	Dorsolateral prefrontal cortex	0.79
29	−65.22	−7.35	27.28	4	Primary motor cortex	0.98
30	67.04	−9.3	17.27	3	somatosensory cortex	1
31	59.3	19.86	19.5	9	Dorsolateral prefrontal cortex	0.79
32	48.7	46.53	12.36	46	Dorsolateral prefrontal cortex	0.61
33	29.74	63.63	13.27	10	Frontopolar area	0.92
34	9.37	70.37	12.07	10	Frontopolar area	0.92
35	−16.19	68.4	13.62	10	Frontopolar area	0.92
36	−38.43	58.91	9.29	10	Frontopolar area	0.92
37	−52.28	39.05	9.49	46	Dorsolateral prefrontal cortex	0.61
38	−60.88	11.92	14.38	44	Pars opercularis Broca’s area	0.73
39	−66.83	−14.27	15.45	43	Subcentral area	0.68
40	62.97	7.33	8.98	6	Pre-motor and supplementary motor cortex	0.9
41	53.89	39.99	4.04	46	Dorsolateral prefrontal cortex	0.61
42	40.63	60.05	0.58	10	Frontopolar area	0.92
43	19.42	70.09	2.84	10	Frontopolar area	0.92
44	−8.28	70.72	0.82	10	Frontopolar area	0.92
45	−28.68	64.9	0.19	10	Frontopolar area	0.92
46	−44.19	54.77	−2.08	10	Frontopolar area	0.92
47	−54.88	34.76	0.43	45	Pars triangularis	0.7
48	−63.53	−1.4	−2.08	22	Superior temporal gyrus	0.46

The fNIRS experiment flow was as follows: upon accessing the research facility, subjects were informed a description of the experiment, given a permission form, and instructed to take a seat. In a line with the tibial nerve, the 2 mucilaginous electrodes of the stimulator were inserted roughly three fingers above the medial malleolus. Patients had fNIRS electrodes placed on their foreheads and then they closed their eyes in a darkened environment. A total of 4 fNIRS scans containing 3 blocks each were completed for each patient. The block design was used: 60 s each for the task and rest periods, and 3 to 5 repetitions of each period. Fifteen seconds of baseline resting data were added before the block to ensure the steady state of the fNIRS signal, and a total of 360 s of data were collected.

**Figure 1:**
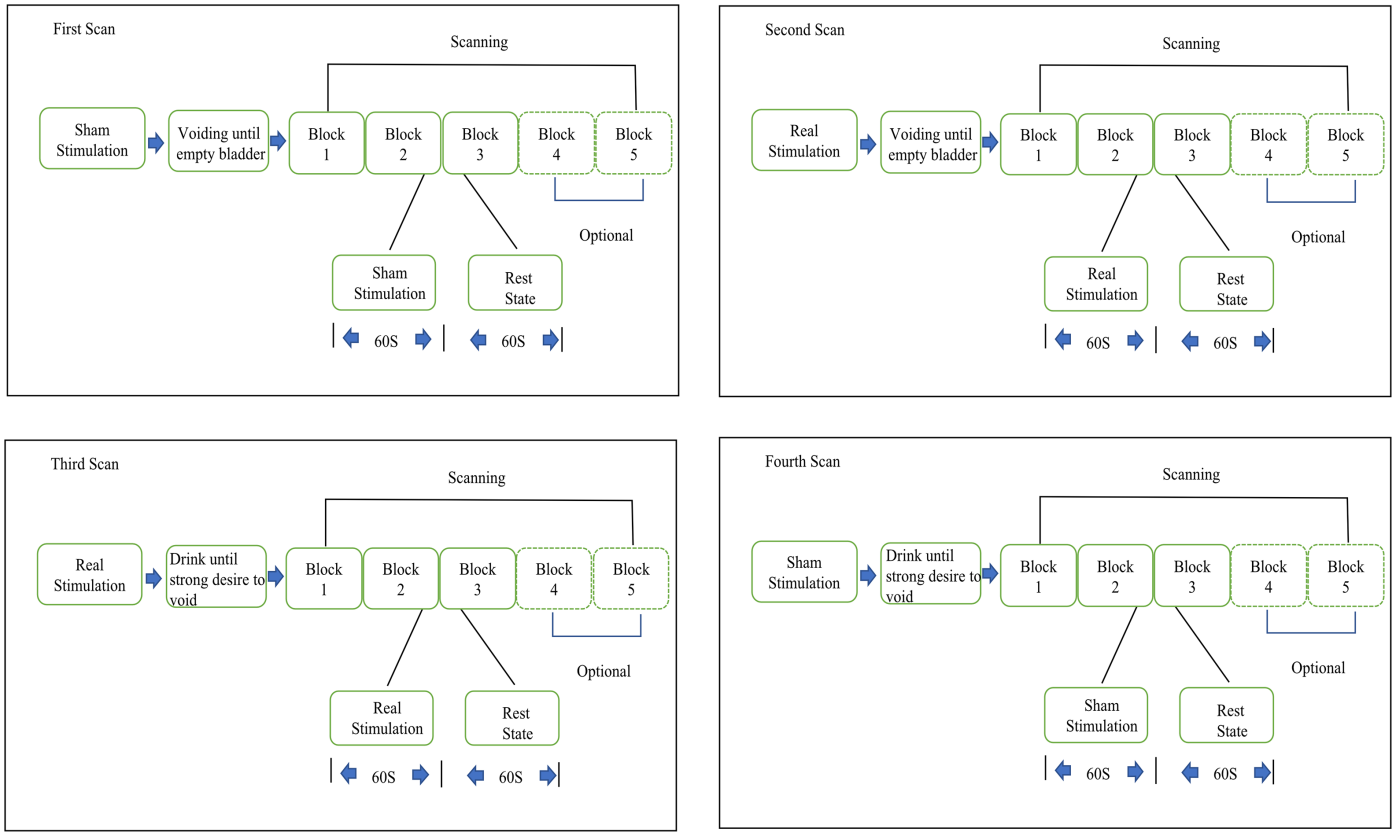
Block diagram of the fNIRS experimental design.

During the task period, patients used TTNS (General Stim, Inc., Hangzhou, Zhejiang, China) on the right lower limb with parameters of 20 Hz frequency, a 0.2-millsecond pulse width, and a 30-milliamp (mA) stimulatory current. The initial scan was obtained with sham stimulation (using the same TTNS device and parameters but the power of the device was off which inducing no stimulation effects) with an empty bladder and a second time with verum stimulation with an empty bladder. The third fNIRS scan was conducted on the subjects after they were given water until they exhibited a strong desire to void (SDV) without being concerned about leaking. Because OAB patients cannot maintain urine storage for a long period with SDV, patients needed to void after the third fNIRS scan. After a period of rest, the patients then were given water until they exhibited SDV state. The fourth scan with sham fNIRS scan in the SDV state was performed (Figure 1). The block design provides many advantages, including the reduction of the need for human involvement and the suppression of oscillations in data that are not relevant (Sato et al., 2007).

### fNIRS equipment

**Figure 2:**
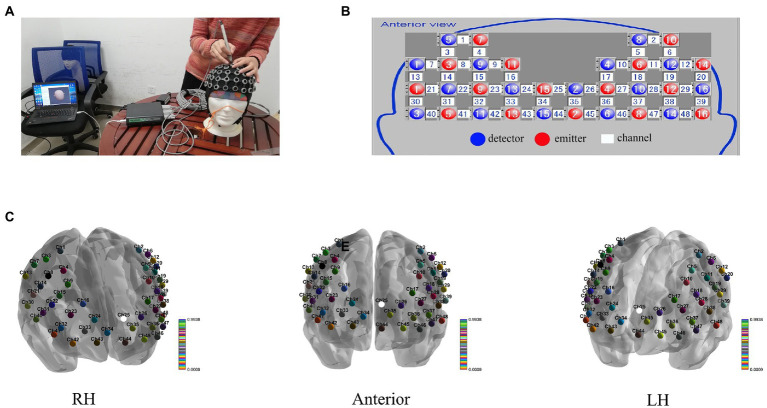
**(A)** Transcranial mapping navigation to locate areas of interest, **(B)** the sensor array, and **(C)** the 48 channels’ 3D MNI coordinates.

To monitor the variations in HbO and HbR in the venous blood of the cortex cortical areas, a two-channel fNIRS topography apparatus (Shimadzu Co.) was utilized. Light-NIRS is capable of capturing hemodynamic responses by concurrently irradiating near-infrared light in three wavelengths (780, 805, and 830 nanometers) using optical cables. The probe system, consisting of a skull cap with 16 near-infrared light emitters, 16 detectors, and 48 channels, was placed on the frontal lobe, with the lowest probes located along the Fp1-Fp2 line (Okada and Delpy, 2003)(Figure 2). A 3D digitizer (Patriot; Polhemus) was used to generate the position information of total circuits and evaluated utilizing NIRS_SPM to get the Montreal Neurological Institute (MNI) coordinates and the possibility of connected brain areas in the Brodmann area (BA) atlas (Jiang et al., 2020; Xu et al., 2020). Channel location details are shown in Table 1.

### fNIRS data analysis

A MATLAB toolbox (Hou et al., 2021) was used to perform data preprocessing and visualize the results. To guarantee a steady signal, the fNIRS data were trimmed by the initial and final 15 s. We used a first-order detrend to get rid of the sluggish time-based fluctuations (Racz et al., 2018). The temporal derivative distribution repair method was used for motion correction (Fishburn et al., 2019). In addition, artifacts were removed by band pass filter limiting the data between 0.008 and 0.08 Hz (Bulgarelli et al., 2020). In this investigation, we focused only on variations in HbO since that signal has been shown to be more sensitive than HbR in detecting differences in regional cerebral blood circulation (Fu et al., 2014). After fNIRS data preprocessing, the individual-level analysis may be performed using the mass univariate statistical approach based on GLMs. For the statistical analysis, the steps listed below were used. To begin, creating a GLM that models the observed hemodynamic signal as a linear mixture of target regressors, unwanted variables, and an error term. Constructing the reference time series representation from task variables using the canonical hemodynamic response function defined in SPM is required for GLM definition. Then, the estimation of GLM parameters on a channel-by-channel basis, which fined the activation beta value for each experimental condition. In the end, utilizing contrast vectors from the pre- and post-stimulus as the input for subsequent group-level inference, the condition-wise effects were calculated. Paired t-test was used for the group-level analyses, corrected by false discovery rate (FDR, *p* < 0.05) (Hou et al., 2021).

### Statistical analyses

We used GraphPad Prism software to conduct statistical analyses. Descriptive data were descripted as mean ± SD or median (25th to 75th percentile) in accordance with the assumption of the normality of the data. Student’s t-test or Wilcoxon test was done on paired continuous variables according to the kind of distribution. *p* < 0.05 was regarded statistically significant.

## Results

Eighteen right-handed women with OAB who elected TTNS treatment were included in our research. Prior to the experiment, none of the patients received TTNS. Sixteen patients were treated successfully, while two were unsuccessfully treated. Table 2 shows baseline statistics of successfully treated patients. Among the patients, 4 had OAB-wet and 11 had nocturia.

**Table 2:** Baseline demographic and clinical characteristics of successfully treated patients.

	OAB (*n* = 16)
Age, years	36.25 ± 16.04
BMI, kg/m^2^	22.34 ± 3.02
OAB Type	
*OAB-Dry*	12 (75%)
*OAB-Wet*	4 (25%)
Duration of OAB symptoms, years	3.97 ± 2.18
Handedness	Right-handed

### Comparison of voiding data before and after TTNS treatment

The clinical parameters showed varying degrees of substantial improvement relative to pretreatment levels (Table 3). The average daily number of micturition, incontinence episodes, and urgency score were decreased from 13.40 ± 2.23 to 7.79 ± 1.22, 6.50 (0.75 to 13.75) to 4.17 (0.00 to 8.59), and 3.62 (0.90 to 3.92) to 2.00 (0.00 to 2.70), respectively. The mean voiding volume was increased from 125.80 ± 33.42 mL to 149.00 ± 36.74 mL. The OABSS, QoL, and PPBC were reduced from 6.06 ± 2.52 to 3.94 ± 2.86, 4.63 ± 0.96 to 2.56 ± 1.79, and 4.50 ± 1.10 to 2.88 ± 1.54, respectively.

**Table 3:** Clinical parameters before and at completion of TNS treatment.

Parameters	Pre-treatment	Post-treatment	*p*-value
Micturition frequency daily	13.40 ± 2.23	7.79 ± 1.22	<0.05
Mean voiding volume (mL)	125.80 ± 33.42	149.00 ± 36.74	<0.05
Number of incontinence episodes per day	6.50 (0.75–13.75)	4.17 (0.00–8.59)	<0.05
Number of Nocturia	1.85 ± 0.85	1.09 ± 0.54	<0.05
Urgency Score	3.62 (0.90–3.92)	2.00 (0.00–2.70)	<0.05
OABSS	6.06 ± 2.52	3.94 ± 2.86	<0.05
QoL	4.63 ± 0.96	2.56 ± 1.79	<0.05
PPBC	4.50 ± 1.10	2.88 ± 1.54	<0.05
PVR	<10 mL	<10 mL	

### Comparison of fNIRS data between sham stimulation and verum stimulation in empty bladder and SDV in successfully treated group

**Figure 3:**
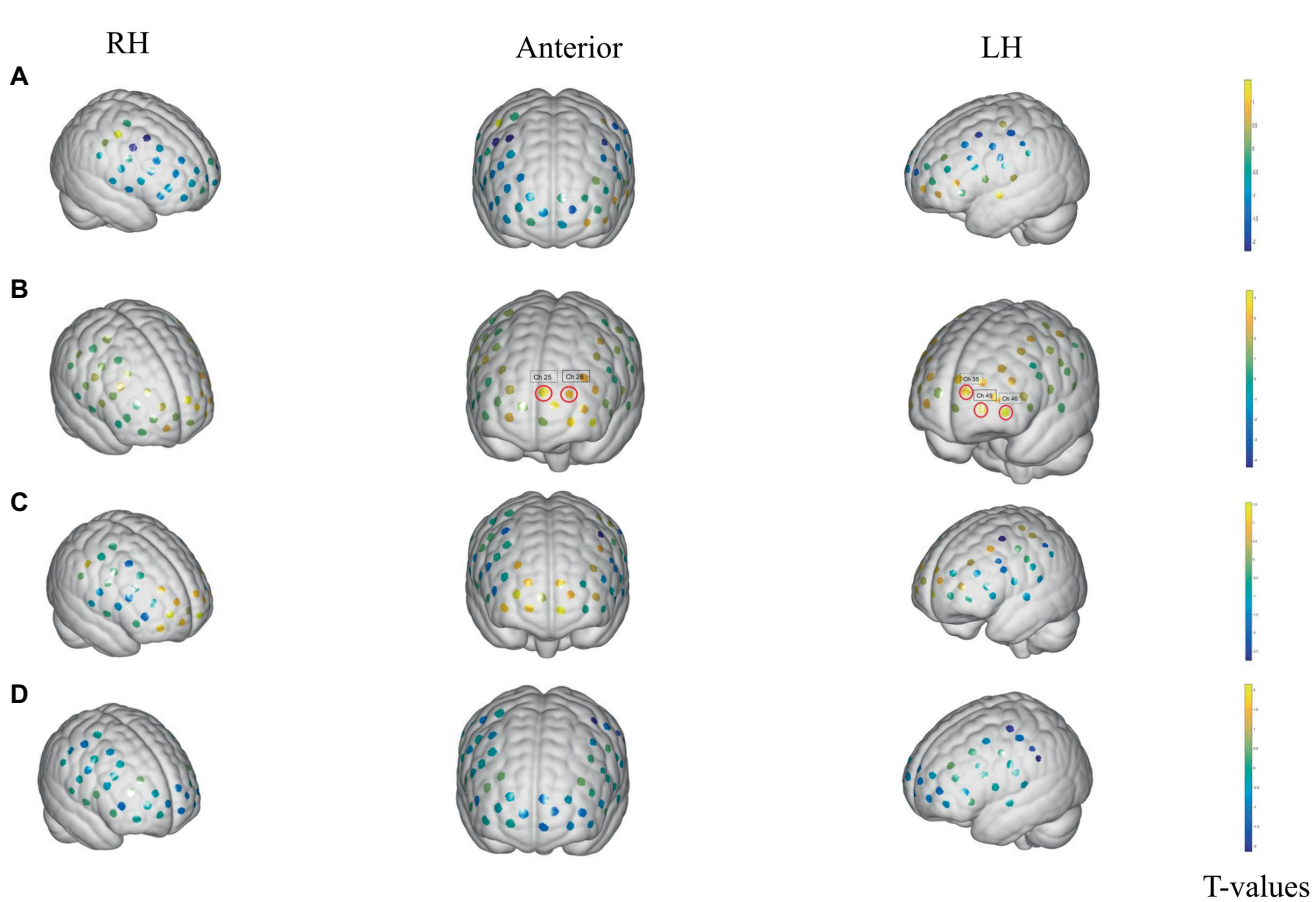
Activation changes of ROI fNIRS data in the empty-bladder and SDV states in successfully treated group. **(A)** Activation of sham stimulation state with empty bladder. **(B)** Activation of verum stimulation with empty bladder. **(C)** Activation of verum stimulation in SDV state. **(D)** Activation of sham stimulation in SDV state. The channels are denoted by the dots. Significance in activation variations is shown by red loops in front of channels (*p* < 0.05, FDR corrected). The colored bar reflects group-level *T*-values. *T* The cool hue represents deactivation, whereas the bright color represents activation.

During the sham stimulation condition, patients with an empty bladder showed no significant changes in any brain regions between the stimulation and rest states. The T-values between the two states are shown Figure 3A. However, in the verum stimulation state, there was significant activation in some brain areas between the stimulation and rest states, such as dorsolateral prefrontal cortex (DLPFC) (BA 9, Chapters 25 and 26), and the frontopolar area (FA) (BA 10, Chapters 35, 45 and 46). The T-values between the two states are shown in Figure 3B. In the SDV state, there were no significant changes in any brain areas both in verum stimulation (Figure 3C) and sham stimulation conditions (Figure 3D).

### Comparison of fNIRS data between sham stimulation and verum stimulation in empty-bladder and SDV states in the unsuccessfully treated group

The unsuccessfully treated patients with both an empty bladder and SDV state achieve no significant changes in any ROIs both in the sham stimulation state and the verum stimulation state. The sample size of the unsuccessfully treated group was only two, and a larger sample is needed to verify the results.

## Discussion

This is the very first prospective research to evaluate the central TNS mechanism in OAB patients utilizing fNIRS. We found regional brain activation with an empty bladder after successful TTNS in women with OAB. Areas activated included the DLPFC, and FA during TTNS. Furthermore, patterns of brain activity differed between women who responded to TTNS and those who were unsuccessfully treated. Different functional neuroimaging devices have been used to explore brain function during urination for some time. As early as 1996, a study based on CT and MRI found that subjects with frontal-lobe lesions showed detrusor hyperreflexia and unrestrained sphincter slackness, resulting in lower urinary tract symptoms (Sakakibara et al., 1996). SPECT and PET technologies have been steadily utilized to neuroimaging during the last several decades due to the fast growth of functional brain imaging technologies (Fukuyama et al., 1996; Blok et al., 1997, 1998; Nour et al., 2000). After that, fMRI and fNIRS were used to investigate the centralized bladder control mechanism that had been predicted. fMRI measures HbR paramagnetism and has exceptional temporal and spatial resolution (Kitta et al., 2015), whereas fNIRS is based on HbO and HbR absorption of near-infrared light and has the benefits of mobility, outstanding temporal resolution, and convenient for clinical use (Jobsis, 1977).

The mechanism of brain function in urination is not still completely understood. Previous studies suggested a functional paradigm for bladder control, including the brain areas such as thalamus, insula, prefrontal cortex (PFC), and periaqueductal gray (PAG) (Griffiths et al., 2005; de Groat et al., 2015). The DLPFC is primarily responsible for executive functions, including the consolidation of information from multiple senses, preservation of focus, and management of goal-directed activity. According to a fNIRS research, the bilateral DLPFC was highly active in the SDV condition, and the greater the urge to urinate, the greater the bilateral DLPFC activation (Matsumoto et al., 2011). Our earlier work demonstrated aberrant DLPFC deactivation in OAB patients, which may relieve DLPFC inhibition on the voiding reflex (Pang et al., 2022).

TNS is a crucial component in the treatment of OAB since it is both effective and less invasive. Previous investigations have offered clues on the potential mechanisms include inhibition of threshold afferent nerve activity (Choudhary et al., 2016), increasing endogenous opioid peptide levels in the central nervous system (Matsuta et al., 2013), and inducing bladder inhibition through cerebral cortex network reconstruction (Finazzi-Agro et al., 2009). During TTNS, brain areas such as the DLPFC (BA 9, Chapters 25 and 26) and the FA (BA 10, Chapters 35, 45 and 46) were activated in the current study. It seems that TTNS could help relieve OAB symptoms by activating brain areas crucial to the voiding reflex. Griffiths et al. (2005) found that OAB patients showed significantly weaker responses to infusion than healthy patients especially in the anterior insula. When the bladder was completely filled, the infusion elicited heightened reactions throughout most of the brain. Still, the reaction in the orbitofrontal cortex was much weaker than it was in individuals with strong control. In this study, OAB patients with an empty bladder achieved significant activation in the BA 9 to 10 areas, compared with the stimulation and rest states. However, they did not achieve activation when patients’ bladders were full. This may be because these brain areas are more activated with a full than an empty bladder, and the difference between activation generated by stimulation and that produced by bladder filling is reduced. Our findings suggest that TNS’s potential primary mechanism for OAB is the normalization of the voiding reflex and the restoration of DLPFC, and FA activation.

This study has limitations. Due to the insufficient size of the patient sample, the findings were not adjusted for the full complement of channels. Furthermore, fNIRS could not monitor the activation alteration of the whole brain cortex and deep brain structures due to the limitations of the detection range imposed by the number and penetration depth of probes. Improved fNIRS technology and analytical techniques may eventually solve this problem.

## Conclusion

TNS has an effect on the brain function of OAB patients who show a clinical response to the treatment. To some extent, it may be how TNS works to alleviate OAB. In subsequent research, fMRI may be used to analyze changes in brain activity associated with clinical responses to medication.

## Data availability statement

The original contributions presented in the study are included in the article/supplementary material, further inquiries can be directed to the corresponding authors.

## Ethics statement

The studies involving human participants were reviewed and approved by the Ethics Committee of the China Rehabilitation Research Center review board. The patients/participants provided their written informed consent to participate in this study.

## Author contributions

LML designed the study. XHL, RF, and XL conducted the research. XHL wrote the manuscript. LML and XL contributed to significant modifications of vital knowledge content. The final version has been authorized by all writers, who accept responsibility for all parts of the work. The final text was reviewed and approved by all writers. All authors contributed to the article and approved the submitted version.

## Funding

The Ministry of Science and Technology of the People’s Republic of China supported this research (2018YFC2002203). The funders had no part in the original study concept, information collection and analysis, publication decision, or manuscript writing.

## Conflict of interest

This study was funded by the authors declare that the research was conducted in the absence of any commercial or financial relationships that could be construed as a potential conflict of interest.

## Publisher’s note

All claims expressed in this article are solely those of the authors and do not necessarily represent those of their affiliated organizations, or those of the publisher, the editors and the reviewers. Any product that may be evaluated in this article, or claim that may be made by its manufacturer, is not guaranteed or endorsed by the publisher.
